# Study on electroless Cu plating quality of in situ TiC_p_

**DOI:** 10.1038/s41598-020-69105-9

**Published:** 2020-07-22

**Authors:** Dongdong Zhang, Yu Liu, Yali Gao, Jinguo Wang

**Affiliations:** 1 School of Mechanical Engineering, Northeast Electric Power University, No. 169 Changchun Road, Chuanying District, Jilin, 132012 P. R. China; 2 Nanchang Institute of Technology, No. 901 Yingxiong Road, Changbei Economic Development District, Nanchang, 330044 China; 3 Key Laboratory of Bionic Engineering, Ministry of Education, Jilin University, Changchun, 130025 P. R. China; 4 Key Laboratory of Automobile Materials, Ministry of Education, Department of Materials Science and Engineering, Jilin University, Changchun, 130025 P. R. China

**Keywords:** Surface assembly, Surface patterning

## Abstract

In situ TiC_p_ was fabricated via combustion synthesis in an Al–Ti–C system. The quality of copper plating was easily observable on the surface of spherical in situ TiC_p_. A study was conducted to assess the influences of the stirring method, plating temperature and particle-to-solution ratio. According to the results, magnetic stirring is an advantageous stirring method. During the plating process, the plating quality reaches the maximum level at 303 K under magnetic stirring. Moreover, uniform and dense plating is achieved when the particle-to-solution ratio reaches 1 g/100 ml. The concentration of solution and ion activity can affect the speed at which Cu^2+^ is attached to the growing core, which plays a significant role in the quality of copper plating.

## Introduction

It is important to study the interface problems, structural characteristics, theoretical models and calculations of metal materials^1–6^. In particular, interface wetting and electroless plating have been widely used to fabricate metallic films on dielectric surfaces. As science and technology have advanced rapidly in recent years, the techniques applied for the production of ultrathin and uniform films have become increasingly important. At present, the deposition process of electroless plating plays an equally significant role in fields such as microelectronics, aerospace technology, automobiles, and mechanical engineering. In recent years, electroless plating has been extensively applied in material science to address surface wettability.

Due to their excellent conductivity, copper and copper alloys have been commonly applied in structural and functional materials such as electrical-resistance welding electrodes and wire^7–10^. Nevertheless, the service life of copper parts tends to be severely reduced because of a combination of high heat loss, low strength, low hardness and poor wear resistance^11,12^. As revealed by recent research, the dispersion of secondary particles in a metal matrix is effective at improving the strength of the material at room and elevated temperatures^13–15^. This concept provides an effective solution for reducing the elevated temperature of copper and copper alloys. As one of the most commonly used reinforcing phases, TiC_p_ is characterized by low density (4.93 g/cm^3^)^16,17^, a high melting point (3,067 °C)^18,19^, high hardness (2,800 HV)^20,21^ and a high diffusion coefficient (8.0–8.6 10^–6^ k^−1^)^22^. Despite these traits, copper and various ceramic particles are considered nonwetting systems^23^. Therefore, a critical technology applied in the fabrication of TiC_p_-reinforced Cu matrix composites is suitable to resolve the nonwetting problem arising between TiC_p_ and the Cu matrix.

**Figure 1:**
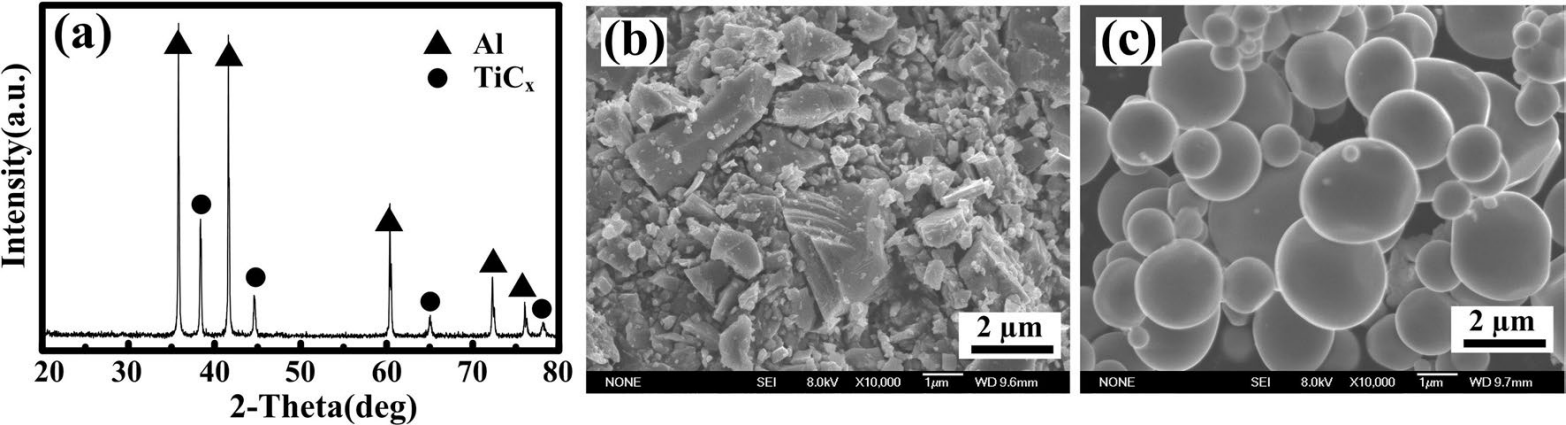
(**a**) XRD pattern of Al-Ti-CNTs composite after combustion synthesis, (**b**) morphology of purchased TiC_p_ and (**c**) morphology of TiC_p_ extracted from TiC_p_/Al composites.

As a sort of autocatalytic oxidation–reduction (REDOX) reaction, electroless plating exhibits various advantages such as high stability, a wide range of working temperatures and ease of operation. In addition, the compact copper layer and excellent binding force lead to a high bonding strength, which makes electroless plating applicable to all kinds of metal and nonmetallic surfaces^24–27^. At present, many electroless plating studies have focused on how copper plating particles can impact the properties of composites. By contrast, there are few studies on the quality of particle copper plating. Moreover, TiC_p_ shapes are irregular, and the available size on the market is uneven, as observed using field emission scanning electron microscopy (FESEM), as shown in Fig. 1b. It is widely known that the shape of reinforcing particles can have a significant impact on the properties of metal matrix materials^28^. A starkly different shape of reinforced particles can exacerbate the anisotropy of the composites, adversely affecting the quality and observations of copper plating. As reported, studies of particle plating quality are quite limited concerning the impact on the properties of copper-coated particle-reinforced Cu matrix composites^29–31^, particularly concerning spherical particles.

In this study, TiC_p_/Al composites containing spherical particles were fabricated by means of combustion synthesis and hot press consolidation in an Al-Ti-CNT system. TiC_p_ was extracted from the TiC_p_/Al composites. The quality of particle plating was studied by adjusting the stirring method, solution temperature and particle-to-solution ratio (PTSR). FESEM was applied to observe the quality of particle plating and study the relevant mechanism. The study of electroless plating on the surface of ceramic particles can address the wetting problem between copper and ceramics. These research results can provide significant guidance for the further study and application of particle surface copper plating.

## Results and discussion

Figure 1a shows the XRD results of Al-Ti-CNT composites prepared by combustion synthesis. The diffraction peaks of Al and TiC_p_ can be clearly seen, indicating success in the fabrication of TiC_p_/Al composites. Figure 1b reveals the morphology of the purchased TiC_p_, and Fig. 1c presents the morphology of the TiC_p_ extracted from TiC_p_/Al composites. As shown in the figure, the purchased TiC_p_ has an irregular shape, and the in situ TiC_p_ extracted from TiC_p_/Al composites has a spherical shape with an average size of 1.45 µm. Apparently, the research value of in situ TiC_p_ on copper plating is higher compared to the purchased TiC_p_.

**Figure 2:**
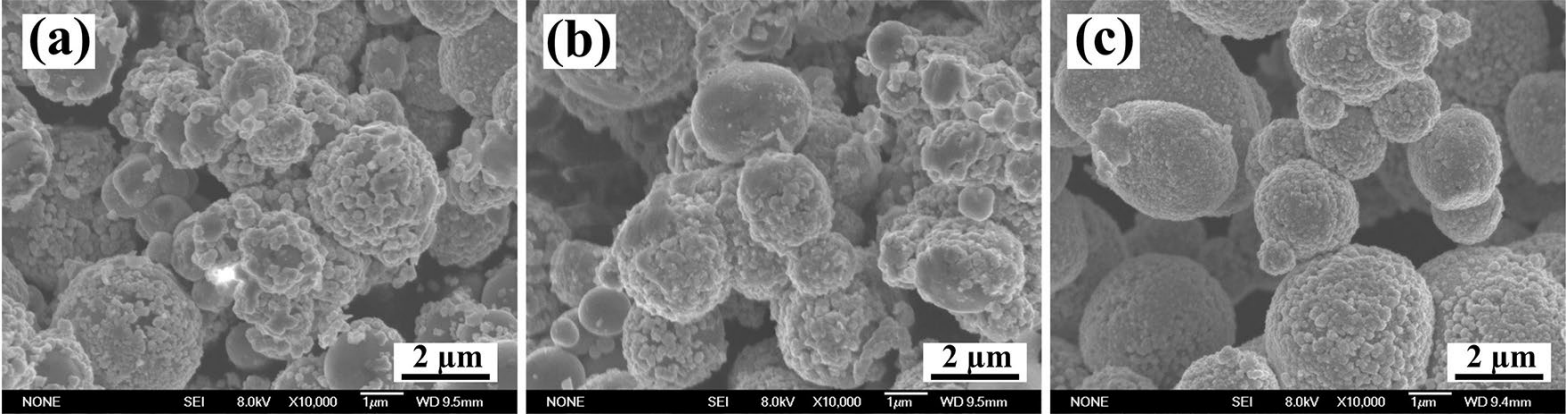
Morphologies of plating coating TiC_p_ with various stirring method (**a**) manual stirring (**b**) ultrasonic stirring (**c**) magnetic stirring.

With no changes to other experimental conditions, a study was conducted on the impact of manual stirring, ultrasonic stirring and magnetic stirring. The morphologies of copper plating TiC_p_ obtained using various stirring methods are illustrated in Fig. 2, which clearly indicates that the plating on TiC_p_ is loose and nonuniform when manual stirring with a glass rod and ultrasonic stirring are chosen as the stirring method for the electroless plating process, as shown in Fig. 2a and b. However, the plating on TiC_p_ is dense and homogenous as a result of magnetic stirring, as shown in Fig. 2c. These results demonstrate that magnetic stirring is more stable than the manual stirring method and is capable of providing a stable ionic environment for electroless copper plating. The concentration of ions in solution is shown to be relatively uniform. The solution provides the same conditions for ion deposits on the surface of particles, and the surface of copper plating becomes smoother. In addition, ultrasonic stirring is effective in making the concentration of ions uniform in the plating solution. Nevertheless, it strips the deposited copper off the platting surface to a certain extent, thus resulting in the deterioration of coating quality. Therefore, magnetic stirring was used as the primary stirring method in the following studies.

**Figure 3:**
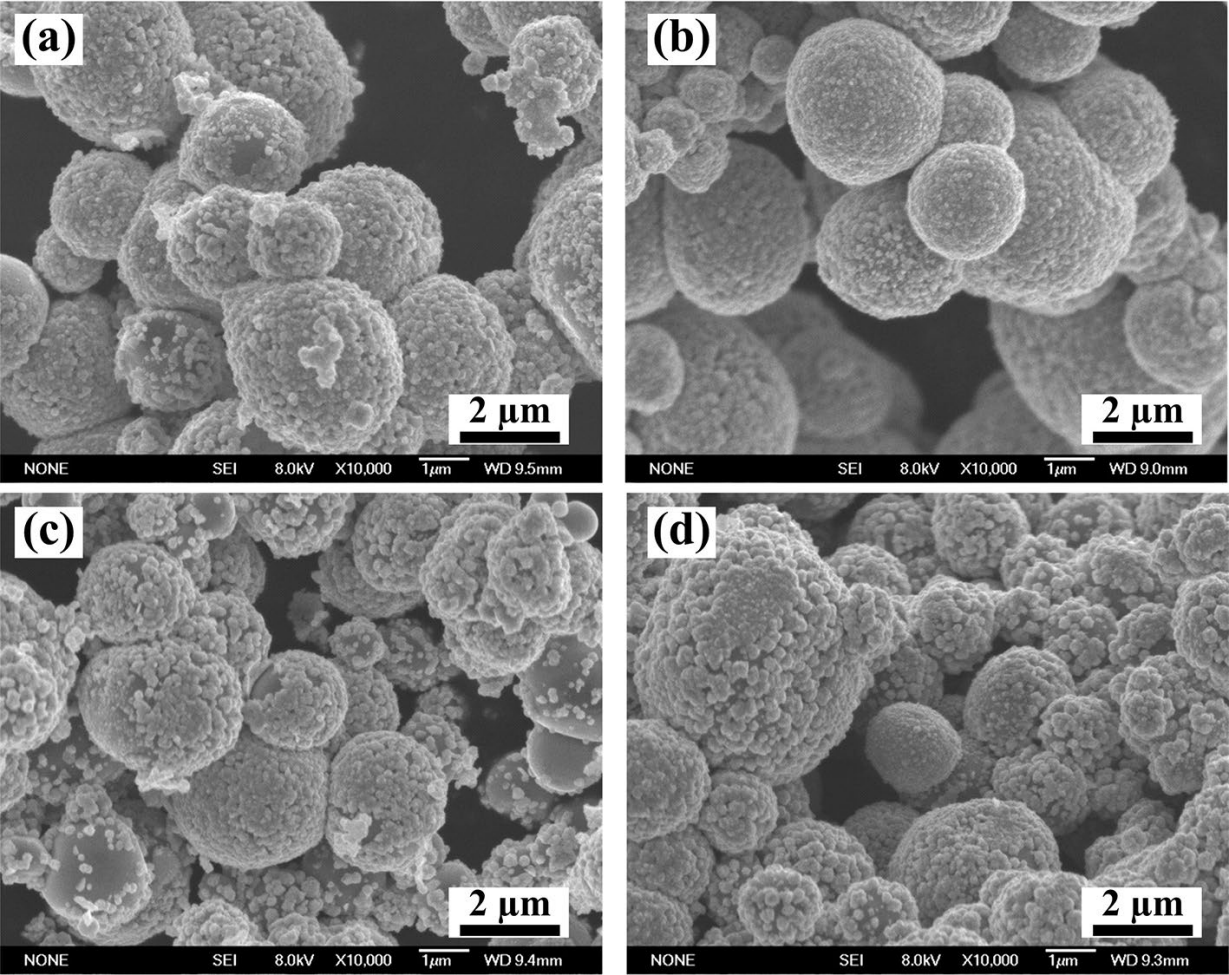
Morphologies of plating coating TiC_p_ at various temperature (**a**) 298 K (**b**) 303 K (**c**) 308 K and (**d**) 313 K.

Figure 3 presents the morphologies of plating on TiC_p_ at experimental temperatures of 298 K, 303 K, 308 K and 313 K. As seen from this figure, the plating is dense and homogenous when the temperature settles at 298 K and 303 K. There are a large number of copper particles formed on the plating surface of coated TiC_p_ at 298 K, which suggests that not all of the copper ions in the solution agglomerate on the surface of TiC_p_ after the REDOX reaction. Instead, some of them conglomerate into particles on the plating. As the temperature increases, the pace of growth increases for the plating. It is denser and more homogenous when the temperature reaches 303 K. When the temperature further increases to 308 K and 313 K, the plating becomes loose and coarse, as shown in Fig. 3c and d. Meanwhile, the plating contains large copper particles and shows a large gap at 313 K. When the temperature of the solution is low, the ions in the solution are sluggish, and Cu^2+^ tends to deposit nearby during the REDOX process, resulting in dense copper plating and the formation of Cu particles. With increasing temperature, the activity of ions in solution is enhanced, the deposition rate rises, and the quality of copper plating is gradually improved. As the temperature continues to rise, the activity of ions in the solution continues to be enhanced, and the deposition rate further rises, thus causing copper plating to be loose. Therefore, the quality of copper plating shows a gradual improvement prior to deterioration with increasing temperature.

The activity of the solution can be enhanced by increasing the temperature, while the enhancement of the solution activity can accelerate the diffusion velocity of Cu^2+^. Moreover, the accelerated diffusion velocity of Cu^2+^ can expedite the pace of deposition and growth for plating. In turn, the increasing pace leads to loose and coarse plating. Therefore, the coating shows the best quality at 303 K for the concentration solution.

**Figure 4:**
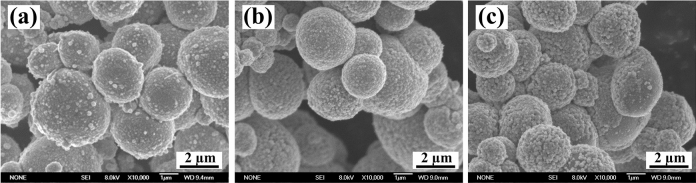
The morphologies of coated TiC_p_ with various PTSR (**a**) 1 g/80 ml, (**b**) 1 g/100 ml and (**c**) 1 g/150 ml.

Figure 4 shows the morphologies of coated TiC_p_ with various PTSRs under magnetic stirring. The PTSR was set to 1 g/80 ml, 1 g/100 ml and 1 g/150 ml. Despite the dense and homogenous plating, the surface roughness of the coating varies with increasing PTSR. The variation in deposition rates leads to varying surface roughness. With the increase in PTSR, the surface roughness of plating declines prior to increasing. The quality of the electroless copper coating is determined by the deposition rate, which is affected by the ion concentration and ion activity in the solution. During the deposition process, the concentration of Cu^2+^ is low, and the level of PTSR is high. The path of Cu^2+^ movement to the particle surface is short, and the growth rate increases for plating, which causes the attachment of Cu^2+^ to the nearby core and its agglomeration into small particles on the plating surface. As the level of PTSR declines, the concentration of Cu^2+^ moderates, and the movement path of Cu^2+^ to the particle surface is extended. In the meantime, the surface of plating becomes uniform, and the number of small particles is significantly reduced. When the level of PTSR reaches 1 g/100 ml, it disappears. The further reduction of PTSR causes the increase of Cu^2+^ content per unit volume and the further acceleration of speed at which the attachment to the coating core occurs, thus improving the growth rate of the coating on the particle surface and reducing the coating densification.

**Figure 5:**
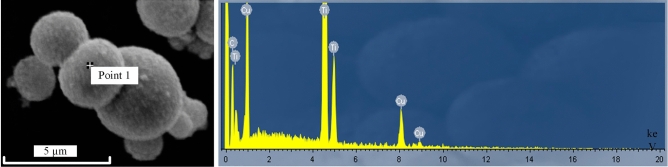
Point analysis of EDS result on the surface of TiC_p_.

The results of the EDS point analysis are shown in Fig. 5, and the chemical composition of the coating is shown in Table 1, which reveals that the chemical composition is elemental Cu, C and Ti. It is also clearly seen that a layer of Cu coating has been formed on the surface of TiC_p_ after electroless plating. As shown in the figure, the surface can be metallized after electroless plating, and success can be achieved in the formation of a layer of Cu on the surface of TiC_p_.

**Table 1:** Chemical composition of the coating on the TiC_p_ particle surface.

Elements	Mass percentage	Atom percentage
C	17.86	47.38
Ti	66.91	46.49
Cu	12.23	6.13
Total	100	

**Figure 6:**
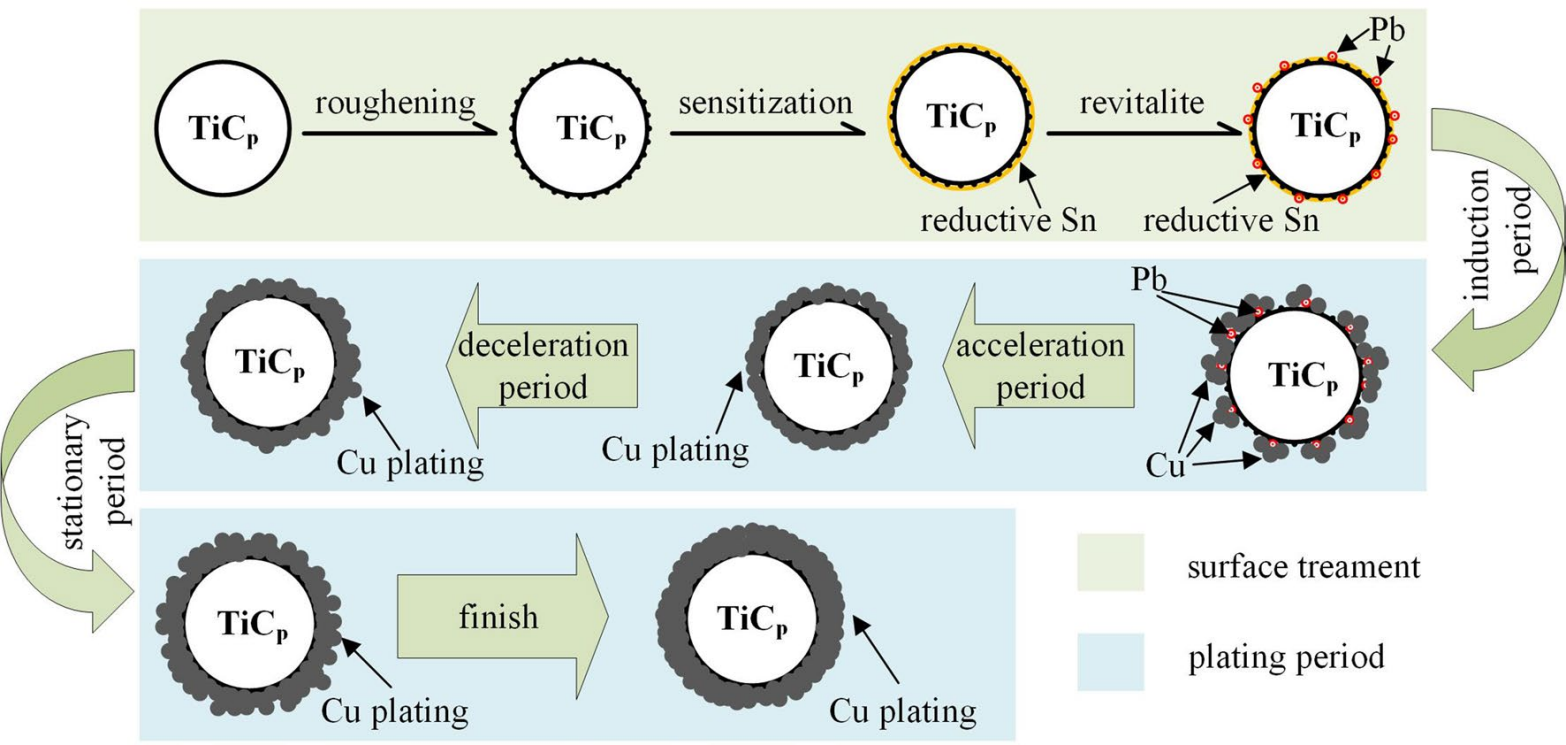
Sketch map of TiC_p_ copper plating cutaway view.

The quality of the Cu coating and its mechanism need to be analyzed. The deposition of electroless plating involves four stages: an induction period, an acceleration period, a deceleration period and a stationary period. It can be seen that there are many active groups that can be enriched on the rough surface of TiC_p_ after the sensitization process. They will adsorb some Pb^2+^ with catalytic activity during the process of revitalized treatment. At the start of the deposition, various ions contained in the plating solution agglomerate near the TiC_p_ to induce the deposition reaction, and the speed of reaction is the lowest. This is known as the induction period. When the induction period comes to an end, a small amount of Cu^2+^ is reduced to Cu on the surface of TiC_p_ with the action of Pb^2+^ by REDOX and begins to nucleate. At this stage, the high activity Pb^2+^ leads to a fast REDOX speed, which is called the acceleration period. When the nucleation of copper on the surface of TiC_p_ is completed, the highly active Pb^2+^ is exhausted, with the deposition of Cu mainly attributed to the activation and catalysis of Cu^2+^ itself. The activity of Cu^2+^ is suppressed relative to Pb^2+^, as a result of which the deposition rate slows down, which is known as the deceleration period. After entering the deceleration period, the deposition rate tends to stabilize gradually, while the coating thickness increases slowly. This stage is called the stationary period. After the completion of REDOX, the process of copper plating is completed. A schematic diagram of the TiC_p_ copper plating process is shown in Fig. 6.

## Conclusion

In this study, an investigation was conducted into the impacts of the stirring method, plating temperature and particle-to-solution ratio on the quality of copper plating. According to the investigative results, magnetic stirring is a stable and continuous stirring method, which is effective in preventing the adhesion between two particles and the variation of concentration. The plating shows the best quality at 303 K under magnetic stirring. The ion activity is moderate, and the plating quality is at its best at 303 K under magnetic stirring. When the level of PTSR reaches 1 g/100 ml, the plating demonstrates the best quality under this condition. Moreover, the concentration of Cu^2+^ is at an appropriate level, the speed of Cu^2+^ attaching to the coating core is moderate, and the plating shows a uniform and dense surface.

## Experimental procedure

### Preparation of spherical TiC_p_

**Figure 7:**
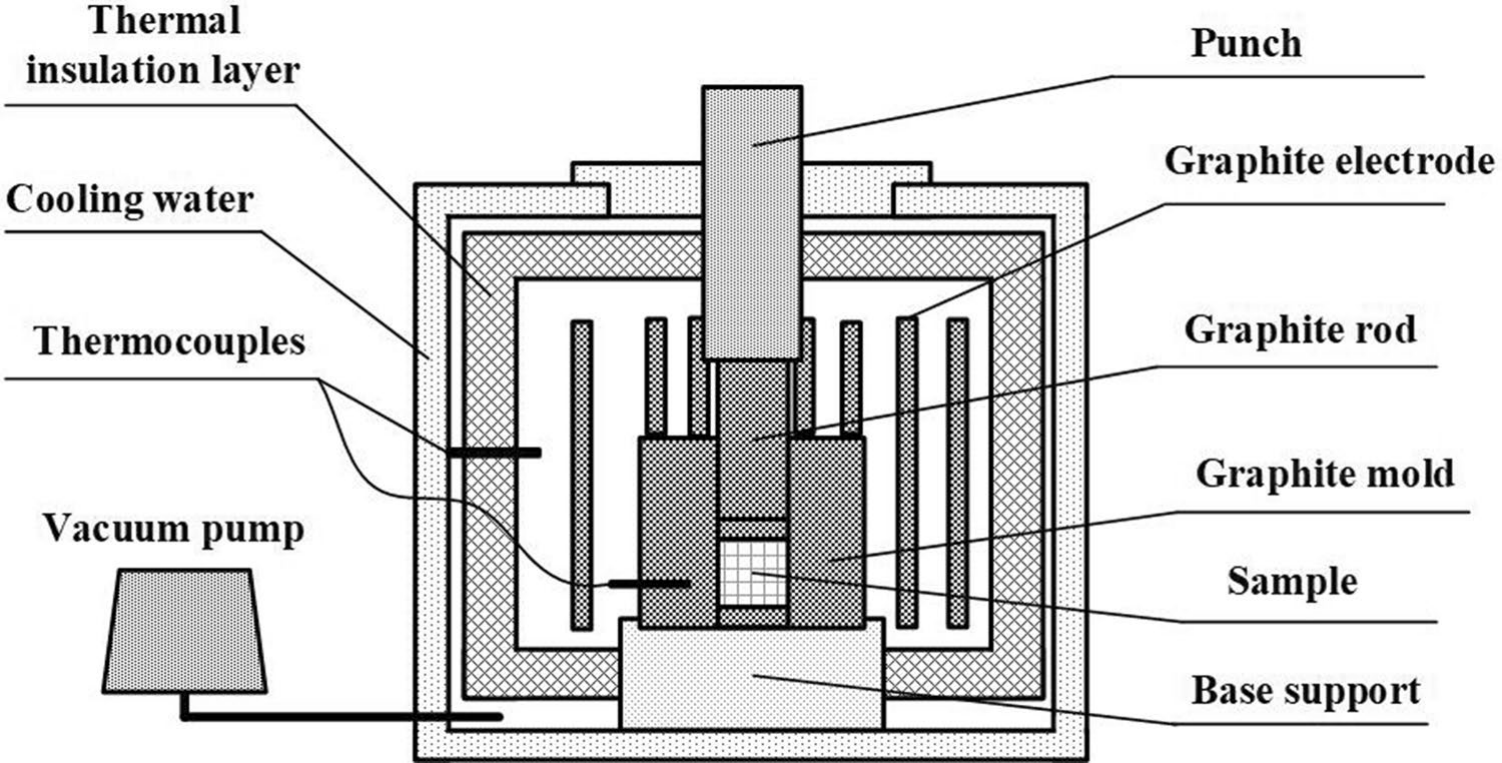
Schematic of self-made vacuum thermal explosion furnace.

The raw materials used in this study consist of various commercial powders, including Al (~ 48 µm), Ti (~ 25 µm), and CNTs (~ 10–25 nm in diameter and ~ 15–100 µm in length). The powders were mixed at a Ti-to-C molar ratio of 1:1 with an Al content of 70 vol%. The powders were mixed sufficiently by ball milling in a container for 24 h and then pressed into preforms with dimensions of 45 mm in diameter and 40 mm in height at room temperature. The combustion synthesis experiments were conducted in a homemade vacuum thermal explosion furnace, as illustrated in Fig. 7. The preforms were first placed in a high-strength graphite mold, which was then placed in the vacuum thermal explosion furnace and heated at a heating rate of 30 °C/min. The heating process was terminated once the temperature, as measured by a thermocouple, showed a rapid increase. When the temperature returned to room temperature, success was achieved in the fabrication of the TiC_p_/Al composite, which was then dissolved into HCl-water solution to remove the Al coating on the surface of the TiC_p_. The extractive TiC_p_ was transferred into a beaker filled with deionized water, which was then placed in the ultrasonic cleaner to clean the surface of TiC_p_ for ten minutes. It was made to stand until the particles sank to the bottom of the beaker and then outwell the top layer of water, with the preceding steps repeated five times. Finally, the beaker was moved into a drying oven to evaporate the water present in the TiC_p_ powders.

### Surface modification of TiC_p_

TiC_p_ surface coarsening was conducted in hydrofluoric deionized water solution at a volume ratio of 1:4, with stirring lasting 15 min. After coarsening, TiC_p_ was cleaned with deionized water 5 times before being placed in a drying oven to evaporate the water contained in the TiC_p_ powders.

The sensitization solution contained hydrochloric and stannous chloride at concentrations of 0.05 ml/l and 0.03 g/l, respectively, and deionized water. TiC_p_ was dissolved into a sensitization solution under stirring for 15 min. After sensitization, TiC_p_ was cleaned with deionized water 5 times and then placed in a drying oven to evaporate the water present in the TiC_p_ powders.

The revitalized solution contained palladium chloride, boric acid, hydrochloric acid at concentrations of 0.0003 g/l, 0.015 g/l and 0.001 ml/l, respectively, and deionized water. TiC_p_ was dissolved into a revitalized solution under stirring for 30 min. After revitalization, TiC_p_ was cleaned with deionized water 3 times before being placed in a drying oven to evaporate the water contained in TiC_p_ powders.

### Copper plating

**Figure 8:**
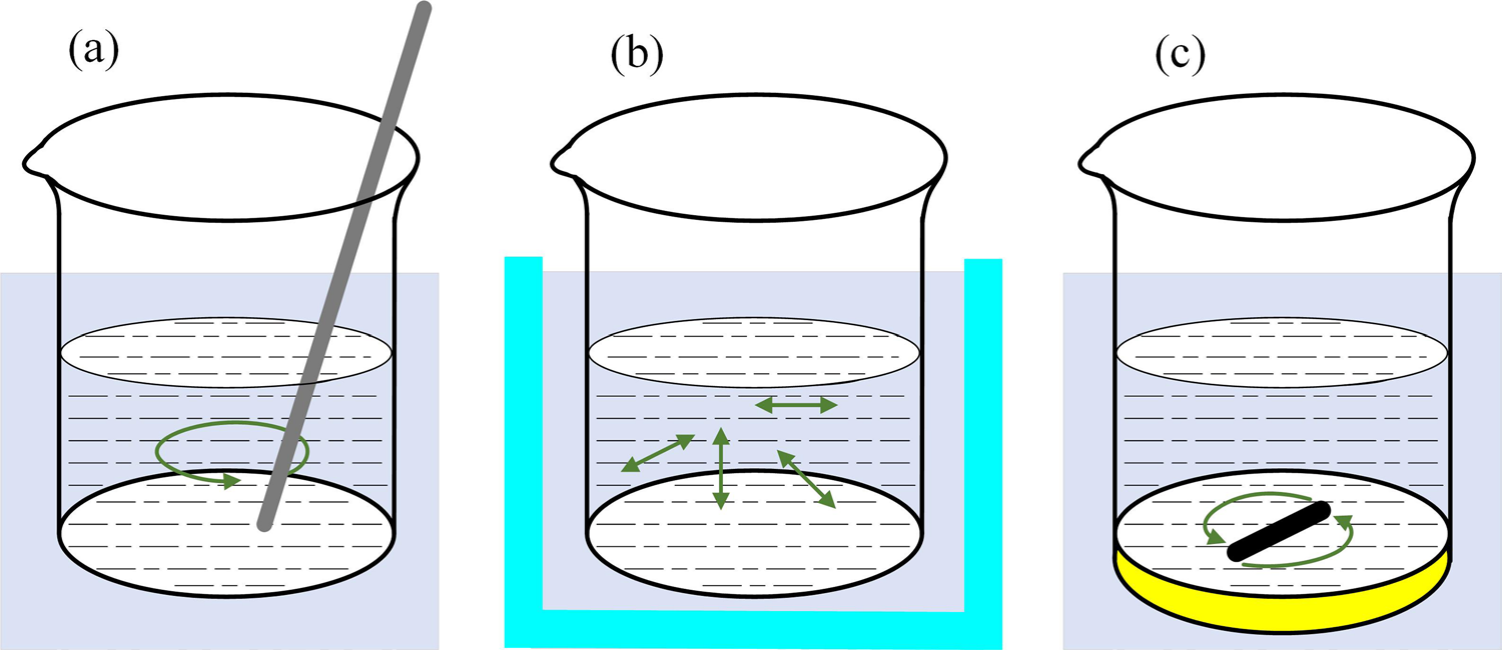
The schematic diagram of various stirring method during the electroless plating process (**a**) manual stirring with glass rod (**b**) ultrasonic stirring and (**c**) magnetic stirring.

The beaker filled with plating solution was placed in a water bath and subjected to heat preservation until the temperature of the solution, as measured by a thermometer, reached the preset level. TiC_p_ was added to the beaker under stirring. During the electroless plating process, manual stirring with a glass rod, ultrasonic stirring and magnetic stirring were adopted as stirring methods. The schematic diagram of various stirring methods is presented in Fig. 8.

### Detection and observation

The phase constitutions of TiC_p_/Al composites were determined by X-ray diffraction (XRD) with Cu Ka radiation at a scanning speed of 4°/min. The morphologies of the extracted TiC_p_ were examined using field emission scanning electron microscopy (FESEM). The element analysis of plating was conducted with the assistance of an energy dispersive spectrometer (EDS).
